# Acute Spinal Cord Syndrome As the Initial Manifestation of Multiple Sclerosis: A Case Report

**DOI:** 10.7759/cureus.98644

**Published:** 2025-12-07

**Authors:** Catarina Pinto Silva, Cristiana Fernandes, Márcia Ribeiro, Dany Cruz

**Affiliations:** 1 Internal Medicine, Hospital Santa Maria Maior, Barcelos, PRT

**Keywords:** demyelinating disease, multiple sclerosis, oligoclonal bands, spinal cord syndrome, transverse myelitis

## Abstract

Multiple sclerosis (MS) typically presents with optic neuritis, brainstem syndromes, or sensory disturbances. Isolated spinal cord involvement as the initial manifestation occurs in fewer than 30% of cases, making early recognition challenging but essential for timely diagnosis and treatment initiation.

A previously healthy 22-year-old Caucasian male presented with an acute onset of mid-thoracic pain followed by progressive sensory deficits below the T10 dermatome and lower limb weakness developing over five days. Neurological examination revealed spastic paraparesis (Medical Research Council grade 3/5), sensory level at T10, and hyperreflexia with bilateral Babinski signs. Magnetic resonance imaging (MRI) of the spinal cord demonstrated two focal T2-hyperintense intramedullary lesions at T1-T2 and T11 levels with gadolinium enhancement. Brain MRI revealed a single periventricular lesion consistent with demyelination. Cerebrospinal fluid analysis showed lymphocytic pleocytosis (11 cells/µL), mildly elevated protein (52 mg/dL), and two oligoclonal bands restricted to cerebrospinal fluid (CSF). The patient received high-dose intravenous methylprednisolone (1 g daily for three days) with significant neurological improvement. At the three-month follow-up, motor strength had improved to grade 4/5 with residual mild sensory deficits.

This case illustrates the diagnostic challenges of MS presenting as isolated spinal cord syndrome. Early spinal MRI and CSF analysis are crucial for accurate diagnosis and prompt therapeutic intervention, potentially improving long-term outcomes.

## Introduction

Multiple sclerosis (MS) is a chronic, immune-mediated demyelinating disorder of the central nervous system (CNS) and a leading cause of neurological disability in young adults. According to the most recent Atlas of MS, approximately 2.8 million people are affected worldwide [1].

Typical initial manifestations include optic neuritis, brainstem syndromes, and sensory disturbances [2,3]. Spinal cord involvement may occur in up to one-third of first presentations but is less frequently isolated, making early diagnosis more challenging [4].

Spinal cord lesions in MS typically appear as short, ovoid, and peripherally located, most often involving less than two vertebral segments [5]. Under the 2017 revision of the McDonald criteria, demonstrating dissemination in space across the brain and spinal cord is essential for diagnosis [6].

Early recognition of spinal cord presentations is critical, as lesion burden strongly correlates with long-term disability and risk of conversion to secondary progressive MS [7,8]. According to the 2017 revision of the McDonald criteria, MS may be diagnosed after a first clinical event when dissemination in space (lesions in at least two typical CNS regions, such as the brain and spinal cord) and dissemination in time (simultaneous presence of enhancing and non-enhancing lesions or cerebrospinal fluid (CSF)-specific oligoclonal bands) are demonstrated [6]. However, when the spinal cord is the sole site of clinical involvement, diagnosis may be delayed due to the rarity and diagnostic overlap with other inflammatory myelopathies. We report a young man who presented with acute spinal cord syndrome as the first clinical manifestation of MS, highlighting the diagnostic challenges, differential considerations, and therapeutic implications.

## Case presentation

A 22-year-old Caucasian male with no prior medical or family history presented with five days of progressive neurological symptoms. He initially developed sharp, band-like thoracic pain at the T6-T8 dermatomes. Within 48 hours, ascending paresthesias appeared in both feet and spread to the umbilical level. By day three, he experienced bilateral lower limb weakness that rapidly progressed, requiring assistance for ambulation.

On admission, his vital signs were stable, and the general physical examination was unremarkable. Neurological examination showed normal strength in the upper limbs (Medical Research Council (MRC) grade 5/5), while the lower limbs demonstrated weakness (MRC grade 3/5) with increased tone and spasticity. A well-defined sensory level was noted at T10, with absent light touch and pinprick sensation below this level. Vibration sense was reduced in the lower extremities, and proprioception was impaired in the toes. Deep tendon reflexes were brisk in the lower limbs (3+ to 4+) with bilateral Babinski signs, while upper limb reflexes were normal. Abdominal reflexes were absent below T10. Cranial nerves were intact, and there were no visual or cerebellar deficits. The patient's gait was spastic and paraparetic, requiring bilateral support. The CSF and serum laboratory investigations are shown in Table 1. 

**Table 1:** Cerebrospinal fluid (CSF) and serum laboratory investigations CSF - cerebrospinal fluid; IgG - immunoglobulin G; CRP - C-reactive protein; ESR - erythrocyte sedimentation rate; MOG - myelin oligodendrocyte glycoprotein

Parameter	Result	Reference range
White blood cells	11 cells/µL (lymphocytic)	<5
Protein	52 mg/dL	15–A45
Glucose	65 mg/dL (serum glucose: 98 mg/dL)	45–80
IgG index	0.85	<0.7
Oligoclonal bands	Positive (CSF-restricted)	Negative
Vitamin B12	485 pg/mL	200–900
ESR	12 mm/h	<20
CRP	0.8 mg/L	<5
HIV serology	Negative	–
Syphilis serology	Negative	–
Aquaporin-4 antibody	Negative	–
MOG antibody	Negative	–

**Figure 1:**
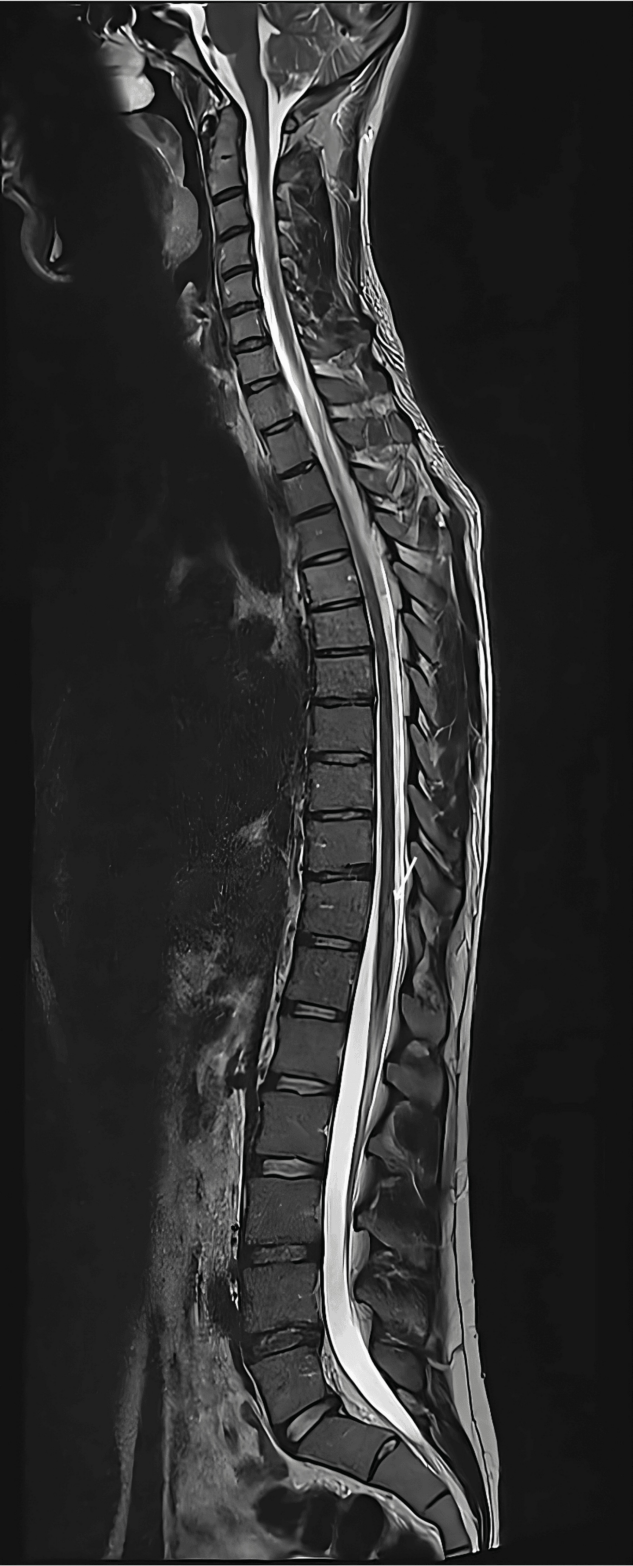
Spinal MRI, sagittal T2-weighted sequence. White arrow highlights focal hyperintense lesion at T11

Spinal MRI revealed two short-segment intramedullary hyperintense lesions on T2-weighted imaging: an ovoid lesion at T1-T2 in the left posterolateral cord and another at T11 in the right posterolateral cord (Figure 1). Both lesions enhanced with gadolinium, without cord swelling or longitudinal extension. Brain MRI demonstrated a small periventricular lesion near the left frontal horn, consistent with demyelination.

A diagnosis of clinically isolated syndrome highly suggestive of MS was made. The patient received intravenous methylprednisolone (1 g daily for three days) followed by a 10-day taper of oral prednisone. Inpatient neurological rehabilitation included physiotherapy, occupational therapy, bladder retraining, and vitamin D supplementation.

At one month, lower limb strength improved to MRC grade 4/5 with partial sensory recovery and independent ambulation using a walking aid (Expanded Disability Status Scale (EDSS) score 3.0) [9]. At three months, strength further improved to 4+/5 with mild residual sensory deficits and an EDSS score of 2.0. MRI surveillance was scheduled, and initiation of disease-modifying therapy was discussed in a multidisciplinary setting.

## Discussion

This case illustrates an uncommon initial presentation of MS in which acute spinal cord syndrome constituted the first and dominant clinical manifestation. While spinal cord involvement is frequent throughout the disease course, it is less commonly the sole initial presentation, occurring in fewer than one-third of patients [4]. Unlike typical longitudinally extensive lesions seen in neuromyelitis optica spectrum disorders (NMOSD) or myelin oligodendrocyte glycoprotein antibody-associated disease (MOGAD), our patient presented with short-segment, peripherally located intramedullary lesions at two separate spinal levels, a pattern that is more characteristic of MS [5].

The detection of a single periventricular brain lesion on MRI is particularly relevant. This finding suggests prior subclinical disease activity and fulfills the criterion for dissemination in space according to the 2017 McDonald criteria when combined with spinal cord lesions [6]. Moreover, the presence of CSF-restricted oligoclonal bands fulfills the criterion for dissemination in time, allowing an earlier diagnosis after a first clinical event. It is therefore plausible that a previous minor central nervous system inflammatory event may have occurred without being clinically recognized by the patient. If the brain lesion had not been present, the diagnosis would likely have remained classified as a clinically isolated syndrome with a lower immediate probability of conversion to definite MS, influencing both prognostic counseling and therapeutic strategy.

Differential diagnoses included NMOSD and MOGAD, which were excluded based on characteristic radiologic patterns, negative aquaporin-4 and MOG antibodies, and the absence of longitudinally extensive transverse myelitis [10,11]. These features reinforce the specificity of the diagnostic workup in distinguishing MS from other inflammatory myelopathies.

Prognostically, spinal cord involvement and early cord atrophy are associated with worse disability trajectories compared with brain lesion burden alone [12-14]. These insights underscore the value of MRI surveillance of both brain and spinal cord, as well as early rehabilitation to maximize functional recovery.

High-dose corticosteroids remain the standard of care for acute inflammatory demyelinating episodes [15,16]. Contemporary evidence supports early use of highly effective disease-modifying therapies in appropriate patients to reduce relapse rates and delay disability accrual [16,17]. Our patient's favorable short-term recovery after corticosteroids and rehabilitation aligns with published experience, while highlighting the need for long-term monitoring and timely initiation of disease-modifying therapy.

## Conclusions

MS should be considered in young adults presenting with acute spinal cord syndromes, particularly when associated with short-segment spinal lesions and inflammatory CSF findings. In this case, the coexistence of spinal cord lesions, a periventricular brain lesion, and CSF-restricted oligoclonal bands fulfilled the 2017 McDonald criteria for early diagnosis. Prompt corticosteroid therapy was associated with significant neurological recovery, supporting early treatment in similar presentations. MRI surveillance and timely initiation of disease-modifying therapy remain essential to reduce the risk of future relapses and disability progression.
